# The impact of COVID-19 on young people’s mental health in latvia

**DOI:** 10.1192/j.eurpsy.2021.794

**Published:** 2021-08-13

**Authors:** N. Konstantinovs, J. Lapa

**Affiliations:** Clinical, Psychotherapy Centre for Adolescents and Young People, Riga, Latvia

**Keywords:** COVID-19, adolescent, young adult, mental health

## Abstract

**Introduction:**

There is an ongoing debate about the impact on mental health associated with Covid-19 pandemics. Some studies have shown an increase in depressive and anxious symptomatology in general population. It has been noted that young people might be among the highest risk populations due to various enviorenmental and developmental influences.

**Objectives:**

To estimate the impact of Covid-19 related restrictions on mental health measures among Latvian adolescents and young adults (14-24).

**Methods:**

We conduct a survey on social media, recruiting 500 participants among the 14-24 age gropup. The survey consists of three parts: 1) sociodemographics; 2) quantitative mental health self-evaluation form; 3) open ended questionaire about the needs and expectations. For statistical analysis we use Excel software and use a regression analysis.

**Results:**

483 participants participated in our survey. The average age was 17.2, 62% was female, 36% male, 2% identified as trans. 52.3% reported decline in their mental functioning and wellbeing in one or several mental health domains (depression, anxiety, addictive behaviours) out of which 13.4% reported significant impairment in a major life area. The support and needs defined by respondents can be divided in three clusters: socialising outside immediate family, psychosocial services, recreational needs.

**Conclusions:**

Confirming to findings in other EU countries, majority of adolescents and young people in Latvia have experienced clinically significant mental health decline during the Covid-19 pandemic. These results can help policy makers in establishing appropriate, needs oriented support in tackling this problem.

